# Experimental and Numerical Investigations of Textile Hybrid Composites Subjected to Low Velocity Impact Loadings

**DOI:** 10.1155/2014/325783

**Published:** 2014-02-25

**Authors:** Gautam S. Chandekar, Ajit D. Kelkar

**Affiliations:** ^1^Department of Mechanical Engineering, Cummins College of Engineering, Pune University, Pune 411052, India; ^2^Nanoengineering Department, Joint School of Nanoscience and Nanoengineering, Greensboro, NC 27401, USA

## Abstract

In the present study experimental and numerical investigations were carried out to predict the low velocity impact response of four symmetric configurations: 10 ply E Glass, 10 ply AS4 Carbon, and two Hybrid combinations with 1 and 2 outer plies of E Glass and 8 and 6 inner plies of Carbon. All numerical investigations were performed using commercial finite element software, LS-DYNA. The test coupons were manufactured using the low cost Heated Vacuum Assisted Resin Transfer Molding (H-VARTM©) technique. Low velocity impact testing was carried out using an Instron Dynatup 8250 impact testing machine. Standard 6 × 6 Boeing fixture was used for all impact experiments. Impact experiments were performed over progressive damage, that is, from incipient damage till complete failure of the laminate in six successive impact energy levels for each configuration. The simulation results for the impact loading were compared with the experimental results. For both nonhybrid configurations, it was observed that the simulated results were in good agreement with the experimental results, whereas, for hybrid configurations, the simulated impact response was softer than the experimental response. Maximum impact load carrying capacity was also compared for all four configurations based on their areal density. It was observed that Hybrid262 configuration has superior impact load to areal density ratio.

## 1. Introduction

Optimization of laminated composites for their desired impact properties has been a major challenge faced by today's composite design world owing to the fact of keeping pace with the rapid advancements in technology and engineering. Among the many applications, 10EG and 10AS4 laminates have their primary applications in aviation, military, body armor, and automobile industries. In the modeling of woven composites under impact, the experimental and the classical mechanics approach seems to be expensive, complicated, time consuming, and highly dependent on many parameters such as the properties of the constituent materials, weave patterns, their orientation, and the manufacturing method. A numerical simulation approach seems to be a promising research and study area as it is fairly accurate, less expensive, and less time consuming.

In the present paper, the work done by Chandekar et al. [1] to compare results using finite element based approach and experimental approach has been extended for 10AS4, Hybrid181, and Hybrid262 laminates. The FE simulations were carried out with a mosaic model approach using commercial FEM software LS-DYNA and the simulation results were compared to the experimental results. The experimental tests were performed using Instron Dynatup 8250 impact test tower. The subsequent sections discus the H-VARTM© manufacturing technique, modeling aspects, impact testing, and results.

In the past, there have been numerous research efforts by many researchers which discuss analytical and numerical simulation approaches. In their paper [2], the authors simulate the ballistic impact on composite laminates, whereas Hosur et al. [3] have experimentally investigated the low velocity impact response to different hybrid configurations. Authors in [4] have studied the low velocity impact response to three curvatures of Carbon Epoxy laminates using quasi-static simulation approach, and Choi [5] has simulated the contact force history in composite sandwich plates subjected to low velocity impact using spring element method in finite element software. In their paper [6], the authors have studied the damage morphology in quasi-isotropic composite panels under variable shape impactors. In [7], the authors have investigated the low velocity impact response to shape memory alloy composite plates using FE simulation. Similarly, in [8], the finite element model to study the impact behavior of preloaded composites panels is presented. The impact on glass reinforced polymers (GRP) using energy model is presented in [9], whereas in [10] the authors have proposed an analytical approach for simulation of low velocity impact using linearized contact law.

One of the major studies in impact analysis of composite structures is bird strike. The bird strike on an aircraft wing leading edge made from fiber metal laminates is studied in [11] using novel SPH finite element approach. Similar analysis was done in [12], where the bird impact on the horizontal tail of a transportation aircraft was presented. In his research publication [13], author proposes a 3D micromechanical analytical model for study of woven hybrid laminates using CLT, whereas [14] presents an analytical model to study the impact behavior of soft armors. Low velocity impact on composite sandwich panels using an assumed strain solid element approach and classical mechanics approach was studied in [15, 16], respectively. In research papers [17, 18] authors discuss the damage tolerance to low velocity impact of laminated composites.

In the past, many researchers have used LS-DYNA as an effective tool in simulating complex problems in classical mechanics and highly complex multidimensional engineering problems. In [19], authors have used LS-DYNA for polymer matrix composite model under high strain rate impact. Similarly LS-DYNA is used to simulate bird strike in [20]. In [21], researchers present novel approach of modeling by reformulating the equations of the collision of two solid bodies incorporating the erosion between them using dynamic coefficients of friction. In their research papers [22, 23] authors simulate the impact damage in double walled sandwich composite structures by incorporating composite ply damage models and interply delamination models. Elder et al. [24] in their research paper compared various de-lamination predictive models such as the linear elastic fracture mechanics model. This was used extensively where the damaged shape of the de-lamination can be predicted and a suitable mesh can be used. In cases where the damage produces irregular de-lamination, this method requires an adaptive mesh approach. The cohesive fracture technique solves some of the limitations of linear elastic fracture mechanics model; however, definitive study of its abilities has not been yet found in the literature.

## 2. H-VARTM© Manufacturing

**Figure 1:**
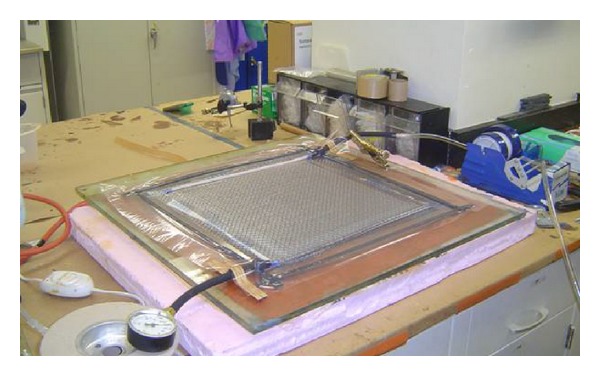
H-VARTM© setup.

Past literature discussed the VARTM (Vacuum Assisted Resin Transfer Molding) process in great detail [25]. Present research uses low cost H-VARTM©, described in [26] as a manufacturing method. H-VARTM© (Heated Vacuum Assisted Resin Transfer Molding) is the process wherein the resin is distributed through the glass or carbon fiber media while controlling heat, pressure, and the flow rate of resin.  Figure 1 shows the picture of H-VARTM© panel being manufactured.

As shown in Figure 1, laminate has been set for curing in an oven with a preset curing cycle. Curing is the process where the laminate is placed in to a temperature controlled oven. In the curing cycle, the temperature is increased by 10°F in steps until the curing temperature is achieved. Then the temperature is maintained at the resin manufacturer's recommended cure temperature. After a predetermined length of time, the temperature is lowered slowly to ambient room temperature and the laminate is removed from the oven.

## 3. Modeling and Property Evaluation

**Figure 2:**
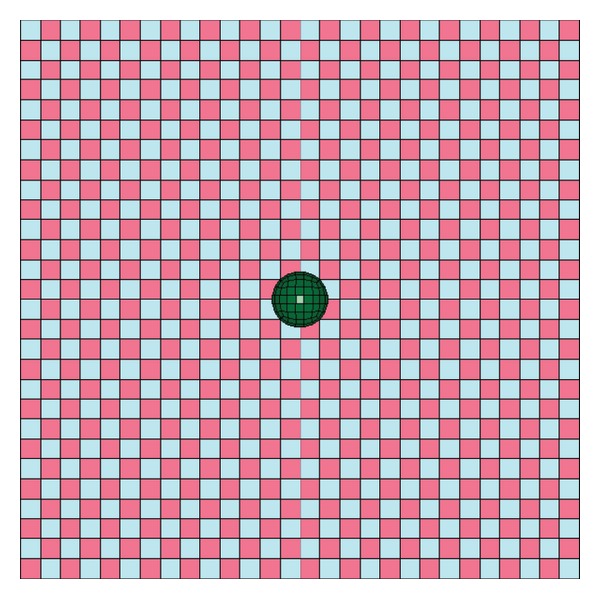
Mosaic model (laminate with impactor).

The modeling of the composite laminate is done using a mosaic fashion as shown in Figure 2. The laminate model was fully developed using the repeating unit. The repeating unit is the smallest unit that repeats in the whole laminate system in *x*-*y* plane. The fabric consists of tows placed during the manufacture of the fabric in two directions: the warp and the weft. In the model, the warp (0°) and the weft (90°) of the composite are represented by a unit cell. The unit cell is created using an 8 node solid element. In the unit cell, the warp (0°) and the weft (90°) are assigned with orthotropic elastic material properties. The resultant properties of the composites unit cell are computed using its constituent's properties, that is, fiber properties and matrix properties together. The unit cell consists of unidirectional fibers impregnated in the epoxy resin, which makes it behave as an orthotropic elastic material making it much stronger in one direction (0°) and weaker in the other (90°) direction. Material model Composite_Damage in LS-DYNA was invoked for the laminate model. The impact load versus time was plotted and the same results were compared with the experimental results (see Figures 9 and 10 and Tables 4 and 5).

There are many possible ways to compute the unit cell properties, some based on experimental study and other on parametric approaches. The analytical method of computing properties is time consuming and the parametric approach requires extensive combinations of tests. Chamis [27] in his research paper presents the simplified equations to compute strengths, fracture toughness, and impact properties based on the micromechanics approach. The same equations were adopted for computing the resultant properties of the 10EG and 10AS4 unit cells.

**Figure 3:**
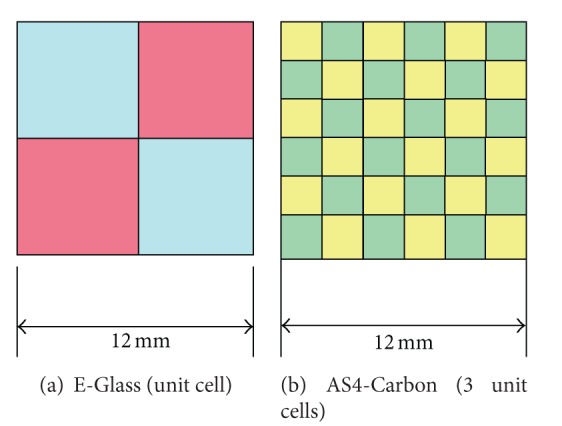
Equivalent mosaic model for E Glass Epoxy and AS4 Carbon laminates.

Figure 3 shows the E-Glass unit cell which corresponds to three unit cells of AS4 Carbon. The mesh size was kept same for E-Glass and AS4-Carbon to maintain node-to-node connectivity.

The impactor head is modeled as a solid sphere discretized using four node quad elements. The steel impactor is assumed to be a rigid ball and is constrained in 5 degrees of freedom (*x* and *y* translation and 3 rotations) allowing its movement only in *z* direction. The effective density equal to 7860 (kg/m^3^), Young's modulus equal to 200 GPa, and Poisson's ratio equal to 0.3 are assigned to the material of the ball.

**Figure 4:**
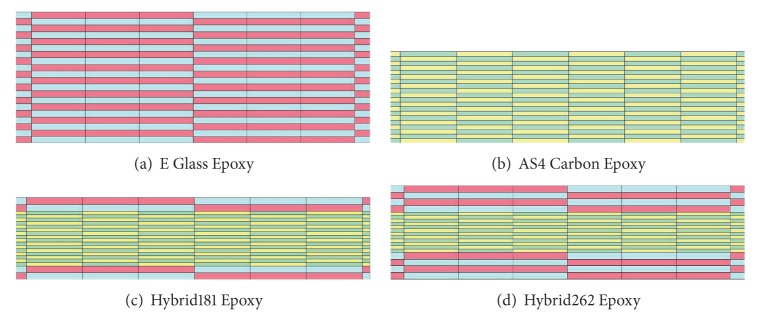
Finite element meshing for various epoxy laminates.

Figure 4 shows the cross section of different ply configurations in FE model. Total number of elements was 110745 and average computational time was 48 clock hours on a single Pentium 4, processor 3.60 GHz, and 3 GB RAM machine.

Table 1 shows the elastic properties of the resin and the fibers used to compute the resultant properties of the laminate unit cell.  Table 2 list the unit cell properties of E-Glass and AS4-Carbon.

## 4. Impact Testing

Impact testing is the experimental procedure where a standard 6′′ × 6′′ square specimen is tested for impact by a standard impactor fitted to the Instron Dynatup 8250 impact tower. The INSTRON DYNATUP 8250 consists of data acquisition system which is connected to the computer for control, data acquisition, and analysis of the impact test load.

**Figure 5:**
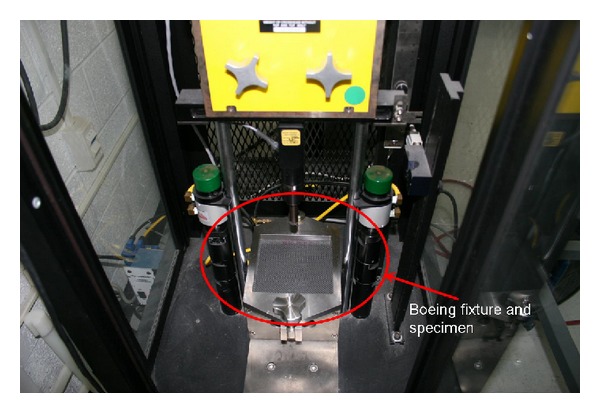
Test coupon clamped in the Boeing fixture.

The impact tower uses an accelerometer for measuring the impact forces. The control of the machine is done through the push buttons on the control pad provided outside the tower, which activates the various electrical and pneumatic controls of the system.  Figure 5 shows the picture of the INSTRON DYNATUP 8250 machine. The impact specimen and the impactor assembly are covered by safety glass with a door provided for specimen access. The impactor consists of a 0.5′′ diameter high strength steel tup, which strikes the specimen surface. The 6′′ × 6′′ square specimen is placed into a Boeing clamping fixture.

A plate is clamped and placed by two stainless steel nuts that are torqued to 27 J applying a fixed boundary condition on all the sides of the plate. The first crack is observed for the matrix cracking which is called an incipient damage and subsequently the impact energy is increased till the laminates are no longer able to resist increase in impact load and is called the upper bound. The range between incipient damage and complete failure is called the progressive damage. Six impact tests were carried out for 10EG, 10AS4, Hybrid181, and Hybrid262 from incipient damage till the complete failure of the laminate at increasing impact energy levels. For each experiment, three specimens were tested and the average value was considered for the analysis.  Table 3 through Table 6 show the details about each experiment, the noted maximum impact load, and impact duration in milliseconds.

**Figure 6:**
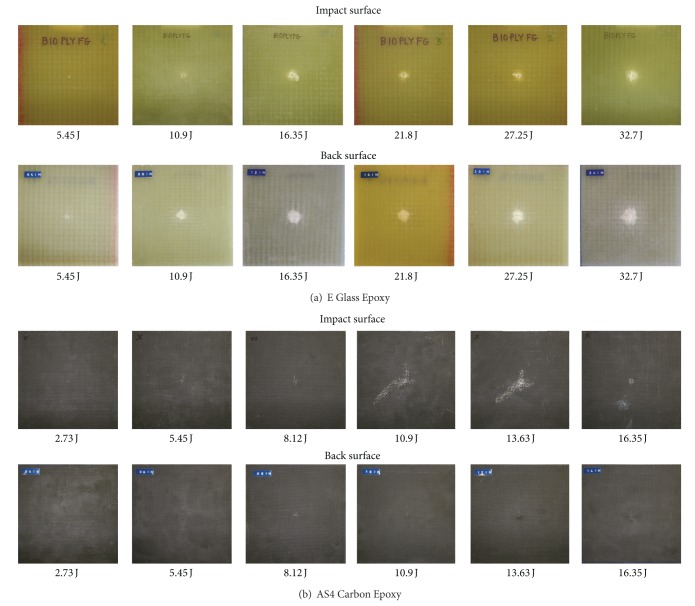
Progressive damage in E Glass AS4 Carbon laminates.

**Figure 7:**
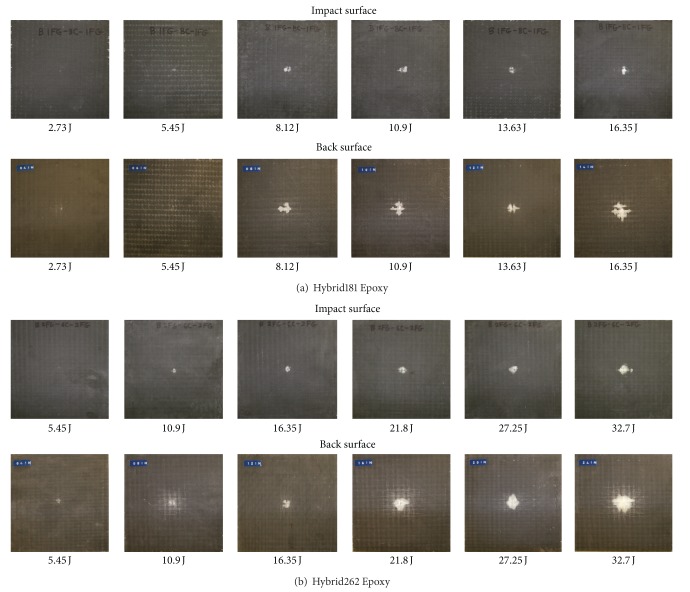
Progressive damage in Hybrid181 and Hybrid262 laminates.

Figure 6 shows the front face and back face picture of the impacted coupons of 10EG and 10AS4 laminates for their progressive damage at six different levels. Similarly Figure 7 shows front and back surfaces for Hybrid181 and Hybrid262 laminates.

In the subsequent section, the impact load versus time is plotted over the range of progressive damage for 10EG, 10AS4, Hybrid181, and Hybrid262. Also, the impact load carrying capacity for each variety is compared.

## 5. Results and Discussion

**Figure 8:**
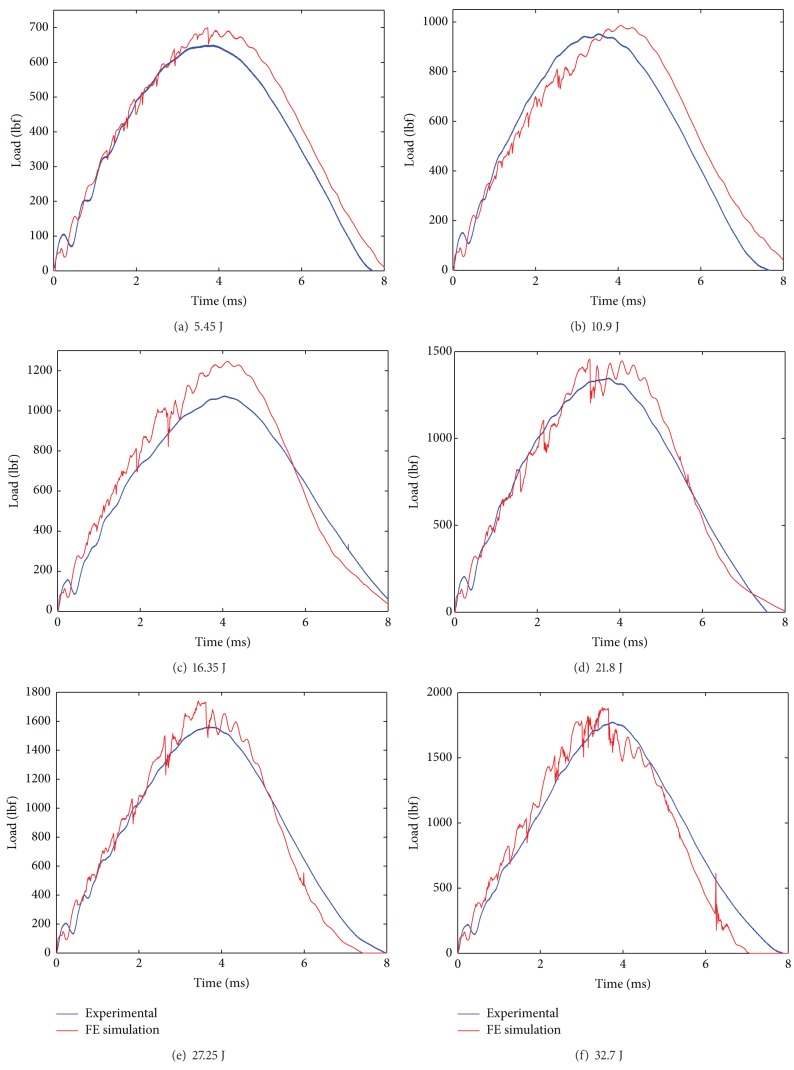
Impact load versus time plots for E Glass/Epoxy laminates at six impact energy levels.

**Figure 9:**
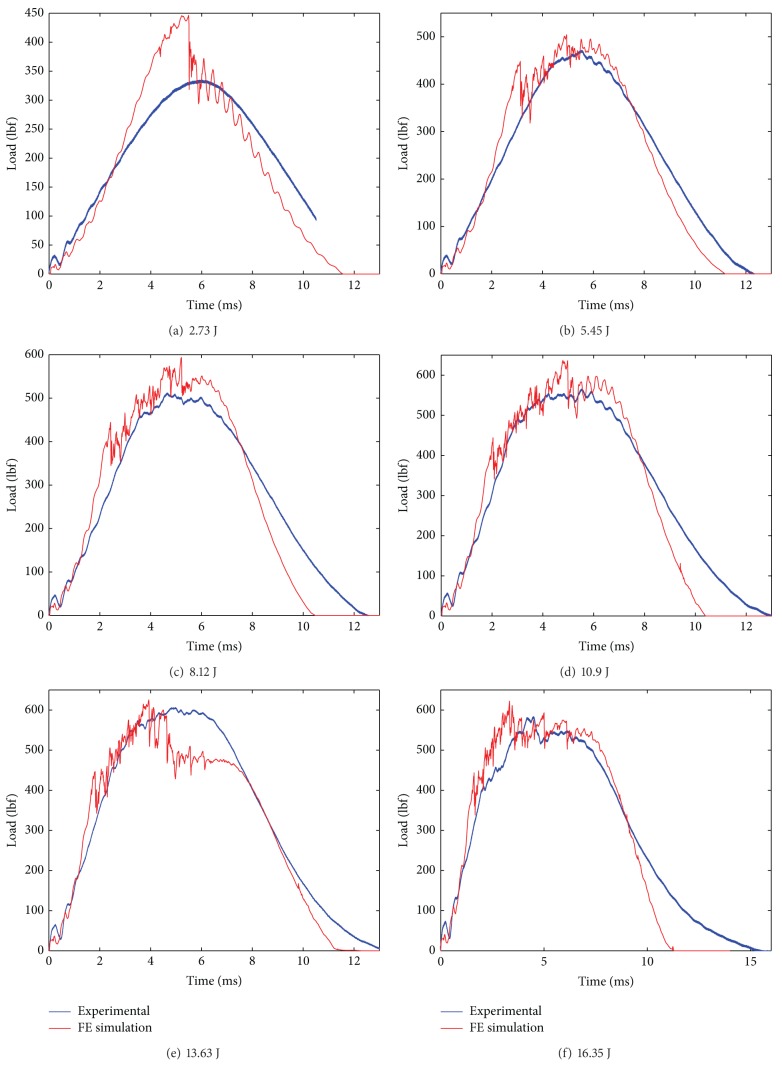
Impact load versus time plots for AS4 Carbon/Epoxy laminates at six impact energy levels.

**Figure 10:**
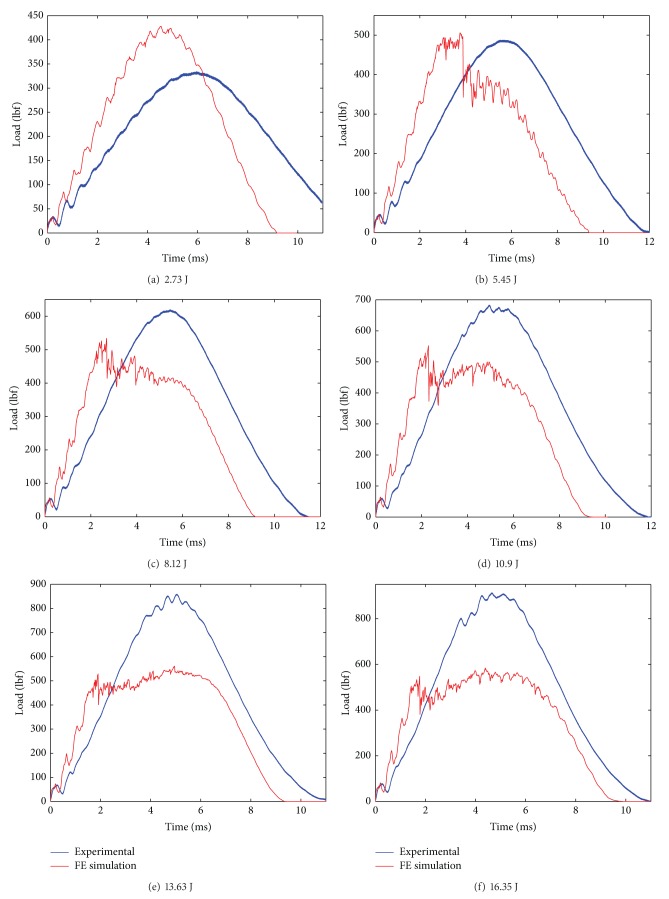
Impact load versus time plots for Hybrid181/Epoxy laminates at six impact energy levels.

**Figure 11:**
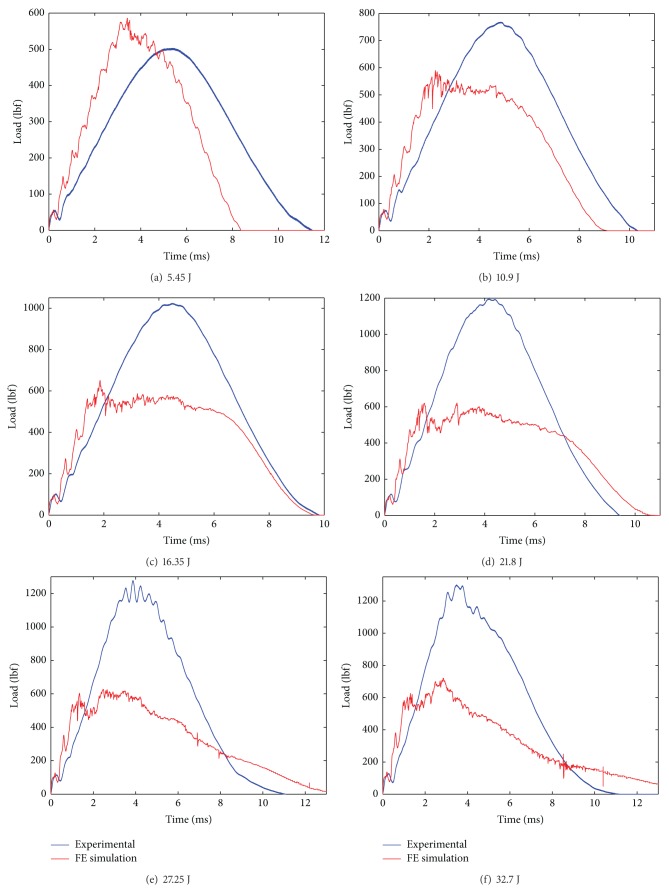
Impact load versus time plots for Hybrid262/Epoxy laminates at six impact energy levels.

The simulated impact load is plotted against the experimental impact load over the range of progressive damage that is at six impact experiments from incipient damage through complete failure. From the impact load versus time plot of 10EG, 10AS4, Hybrid181 and Hybrid262, shown in Figure 8 through Figure 11, it is seen that the simulated impact load is in good agreement with the experimental impact load. It is also observed that, at higher impact energy levels, the maximum simulated impact load is much lower than the experimental impact load. This fact of underpredicting the maximum impact load at higher impact energy levels could be attributed to the fact that the present material damage model uses the maximum stress failure criteria for the failure of the element and once the failure stress is reached, the element fails and does not carry anymore impact load, although in reality some fibers strands do have some load carrying capacity. Also, it is seen that with stress material failure criteria in simulated model, the impact load is on higher side at incipient damage; the reason could be that the plate acts as elastic plate and does not convey the matrix cracking which happens at incipient damage as observed in reality. Thus, the simulated impact response for 10EG, 10AS4, Hybrid181, and Hybrid262 is a little stiffer at incipient damage.

**Figure 12:**
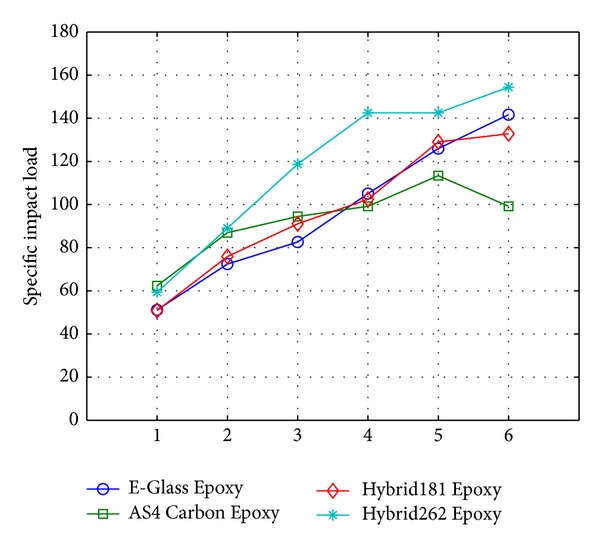
Specific impact load (ratio of load (N) to areal density (kg/m^3^)) versus loading level of progressive damage.

Further, the experimental values of maximum impact load per areal density of the composite, that is, the load factor for 10EG, 10AS4, Hybrid181, and Hybrid262, were plotted over the range of progressive damage and are compared with each other. Also, as seen from Figure 12, the load factor for the Hybrid262 configuration is higher than that of 10EG, 10AS4, and Hybrid181 over the range of progressive damage. The load factor of Hybrid181 is almost similar to that of 10EG. The 10AS4 laminate shows the lowest load factor over the progressive damage.

## 6. Conclusion

In the present study, the progressive impact response of 10EG, 10AS4, Hybrid181, and Hybrid262 was investigated using LS-DYNA and validated with experimental results. The 10EG, 10AS4, Hybrid181, and Hybrid262 coupons were manufactured using low cost H-VARTM© technique. The coupons were experimentally tested for progressive damage using Instron Dynatup 8250 impact test tower. The same progressive damage is modeled using mosaic model and simulated using LS-DYNA. It was found that the simulated response for 10EG and 10AS4 is in good agreement with the experimental response. However, Hybrid181 and Hybrid262, the simulated impact loads, are lesser than the experimental impact loads. Hence, further development of material damage model for progressive material damage is recommended. Also, from the experimental results it is observed that the Hybrid262 is strongest and lightest in its impact resistance among 10EG, 10AS4, and Hybrid181 for the range of progressive damage. Further investigation using ultrasonic C scan would be helpful in understanding the inter-laminar damage quantitatively and qualitatively. Also modeling weave undulation is a prospective research area.

## Figures and Tables

**Table 1:** Constituent's elastic properties.

Property	E Glass Epoxy	AS 4 Carbon Epoxy
Fiber volume fraction *K*_*f*_	0.50	0.50
Matrix voluma fraction *K*_*m*_	0.50	0.50
Elastic modulus for matrix *E*_*m*_ (GPa)	3.45	3.45
Shear modulus for matrix *G*_*m*_ (GPa)	1.28	1.28
Elastic modulus in longitudinal direction *E*_*faa*_ (GPa)	73.08	213.74
Elastic modulus in transverse direction *E*_*fbb*_ (GPa)	73.08	13.79
Shear modulus *G*_*fab*_ (GPa)	30.13	13.79
Shear modulus *G*_*fbc*_ (GPa)	30.13	6.89

**Table 2:** Unit cell properties.

Property ↓	E Glass Epoxy		AS4 Carbon Epoxy	
Fiber orientation →	Warf (0°)	Weft (90°)	Warf (0°)	Weft (90°)
Density (kg/m^3^)	11.74		11.15	
*E*_*a*_ (GPa)	38.27	10.56	125.48	8.07
*E*_*b*_ (GPa)	10.56	38.27	8.07	125.48
*E*_*c*_ (GPa)	10.56	10.56	8.07	8.07
*G*_*ab*_ (GPa)	3.96	3.96	4.13	4.13
*G*_*bc*_ (GPa)	2.45	3.96	2.42	4.13
*G*_*ca*_ (GPa)	3.96	2.45	4.13	2.42
Pr_*ba*_	0.08	0.29	0.02	0.28
Pr_*ca*_	0.08	0.42	0.02	0.47
Pr_*cb*_	0.42	0.08	0.47	0.02

**Table 3:** E-Glass Epoxy experimental results.

Number	Impact level	Drop height	Laminate thickness	Max impact load	Time to reach max load	Impact velocity	Impact energy	Impact duration
Unit →		m	mm	kN	ms	m/s	J	ms
1	1	0.10	5.11	2.77	3.64	1.30	0.72	7.77
2	1	0.10	5.31	2.87	3.65	1.36	1.19	7.79
3	1	0.10	4.90	3.07	3.47	1.38	0.93	7.34
4	2	0.20	5.33	4.32	3.52	1.92	0.93	7.27
5	2	0.20	5.21	3.97	3.58	1.87	1.46	7.85
6	2	0.20	4.90	4.50	3.12	1.90	1.26	6.93
7	3	0.30	4.80	4.93	4.02	2.37	2.59	8.13
8	3	0.30	4.75	4.77	4.02	2.37	3.74	8.34
9	3	0.30	4.90	4.70	4.26	2.38	4.08	8.50
10	4	0.41	4.98	6.09	3.72	2.74	2.76	7.66
11	4	0.41	4.75	6.02	3.56	2.76	4.85	7.47
12	4	0.41	4.83	5.93	3.72	2.76	5.03	7.55
13	5	0.51	4.60	7.36	3.46	3.10	3.54	7.08
14	5	0.51	4.50	6.82	3.80	3.10	6.54	8.02
15	5	0.51	4.62	6.90	3.91	3.13	6.12	8.01
16	6	0.61	4.57	8.12	3.62	3.42	4.30	7.48
17	6	0.61	4.62	7.72	3.78	3.41	9.21	8.06
18	6	0.61	4.95	7.88	3.76	3.42	7.36	7.89

**Table 4:** AS4 Carbon Epoxy experimental results.

Number	Impact level	Drop height	Laminate thickness	Max impact load	Time to reach max load	Impact velocity	Impact energy	Impact duration
Unit →		m	mm	kN	ms	m/s	J	ms
1	1	0.05	2.39	1.51	5.61	0.97	0.95	10.50
2	1	0.05	2.41	1.45	5.94	0.97	1.33	10.66
3	1	0.05	2.44	1.52	5.91	1.00	1.02	11.13

4	2	0.10	2.41	2.15	4.78	1.34	1.38	11.21
5	2	0.10	2.41	2.06	5.55	1.40	1.64	12.41
6	2	0.10	2.36	2.12	5.44	1.34	1.46	12.09

7	3	0.15	2.44	2.40	4.67	1.64	2.87	11.94
8	3	0.15	2.46	2.25	5.54	1.61	2.64	12.61
9	3	0.15	2.34	2.34	4.51	1.66	3.36	12.44

10	4	0.20	2.39	2.35	3.26	1.91	4.65	13.06
11	4	0.20	2.44	2.41	5.46	1.91	4.26	12.85
12	4	0.20	2.39	2.39	5.88	1.96	5.33	13.04

13	5	0.25	2.44	3.19	5.2	2.12	4.98	11.69
14	5	0.25	2.34	2.65	3.66	2.16	6.39	13.39
15	5	0.25	2.39	2.60	3.48	2.14	6.10	12.99

16	6	0.30	2.44	3.53	4.45	2.32	8.41	11.96
17	6	0.30	2.36	2.38	6.02	2.37	8.96	15.10
18	6	0.30	2.36	2.48	6.12	2.41	8.77	15.45

**Table 5:** Hybrid181 Epoxy experimental results.

Number	Impact level	Drop height	Laminate thickness	Max impact load	Time to reach max load	Impact velocity	Impact energy	Impact duration
Unit →		m	mm	kN	ms	m/s	J	ms
1	1	0.05	3.28	2.24	5.61	1.32	0.47	11.39
2	1	0.05	3.07	2.13	5.94	1.34	1.27	12.23
3	1	0.05	3.23	2.18	5.91	1.38	1.74	11.58

4	2	0.10	3.02	2.66	4.78	1.55	1.34	10.87
5	2	0.10	3.25	2.78	5.55	1.68	1.60	11.64
6	2	0.10	3.10	2.82	5.44	1.70	1.83	11.27

7	3	0.15	3.07	2.88	4.67	1.80	2.30	12.12
8	3	0.15	3.23	3.32	5.54	1.92	2.41	11.18
9	3	0.15	3.10	2.99	4.51	1.92	2.93	11.56

10	4	0.20	3.23	3.42	3.26	2.07	3.10	11.6
11	4	0.20	3.20	4.07	5.46	2.17	2.52	10.23
12	4	0.20	3.18	4.10	5.88	2.16	3.17	10.47

13	5	0.25	3.07	4.33	5.2	2.33	3.01	10.26
14	5	0.25	3.28	4.05	3.66	2.39	4.84	10.9
15	5	0.25	3.20	3.98	3.48	2.39	4.83	11.08

16	6	0.30	3.07	4.06	4.45	2.55	6.03	11.56
17	6	0.30	3.23	4.10	6.02	2.74	9.99	12.22
18	6	0.30	3.28	4.06	6.12	2.55	6.03	11.56

**Table 6:** Hybrid262 experimental results.

Number	Impact level	Drop height	Laminate thickness	Max Impact load	Time to reach max load	Impact velocity	Impact energy	Impact duration
Unit →		m	mm	kN	ms	m/s	J	ms
1	1	0.10	3.76	2.40	5.61	1.34	0.53	10.26
2	1	0.10	3.68	2.21	5.94	1.38	1.50	11.39
3	1	0.10	3.76	2.17	5.91	1.37	1.46	11.44

4	2	0.20	3.58	3.54	4.78	1.88	0.98	9.84
5	2	0.20	3.63	3.53	5.55	1.97	2.73	10.35
6	2	0.20	3.78	3.21	5.44	1.84	2.79	10.37

7	3	0.30	3.71	4.92	4.67	2.38	0.89	8.85
8	3	0.30	3.66	4.32	5.54	2.38	4.03	10.02
9	3	0.30	3.78	4.54	4.51	2.41	3.82	9.63

10	4	0.41	3.76	5.55	3.26	2.72	2.98	9.14
11	4	0.41	3.66	5.20	5.46	2.78	6.35	9.49
12	4	0.41	3.61	5.43	5.88	2.76	6.66	9.50

13	5	0.51	3.63	6.95	5.2	3.08	3.97	8.53
14	5	0.51	3.76	5.33	3.66	3.10	14.02	11.03
15	5	0.51	3.66	5.05	3.48	3.11	13.87	11.01

16	6	0.61	3.78	6.48	4.45	3.36	10.45	9.75
17	6	0.61	3.66	5.52	6.02	3.42	18.75	11.37
18	6	0.61	3.61	6.02	6.12	3.38	17.00	10.37

## References

[1] Chandekar GS, Thatte BS, Kelkar AD (2010). On the behavior of fiberglass epoxy composites under low velocity impact loading. *Advances in Mechanical Engineering*.

[2] Silva MAG, Cismaşiu C, Chiorean CG (2005). Numerical simulation of ballistic impact on composite laminates. *International Journal of Impact Engineering*.

[3] Hosur MV, Adbullah M, Jeelani S (2005). Studies on the low-velocity impact response of woven hybrid composites. *Composite Structures*.

[4] Huang C-H, Lee Y-J (2005). Quasi-static simulation of composite-laminated shells subjected to low-velocity impact. *Journal of Reinforced Plastics and Composites*.

[5] Choi IH (2006). Contact force history analysis of composite sandwich plates subjected to low-velocity impact. *Composite Structures*.

[6] Farooq U, Gregory K (2009). Finite element simulation of low velocity impact damage morphology in quasi isotropic composite panels under variable shape impactors. *European Journal of Scientific Research*.

[7] Meo M, Antonucci E, Duclaux P, Giordano M (2005). Finite element simulation of low velocity impact on shape memory alloy composite plates. *Composite Structures*.

[8] Mikkor KM, Thomson RS, Herszberg I, Weller T, Mouritz AP (2006). Finite element modelling of impact on preloaded composite panels. *Composite Structures*.

[9] Mouring SE, Louca L, Levis W Structural response of impact-damaged composite panels.

[10] Choi IH, Lim CH (2004). Low-velocity impact analysis of composite laminates using linearized contact law. *Composite Structures*.

[11] McCarthy MA, Xiao JR, McCarthy CT (2004). Modelling of bird strike on an aircraft wing leading edge made from fibre metal laminates—part 2: modelling of impact with SPH bird model. *Applied Composite Materials*.

[12] Ubels LC, Johnson AF, Gallard JP, Sunaric M (2003). Design and testing of a composite bird strike resistant leading edge. *Report National Aerospace Laboratory*.

[13] Donadon MV, Falzon BG, Iannucci L, Hodgkinson JM (2007). A 3-D micromechanical model for predicting the elastic behaviour of woven laminates. *Composites Science and Technology*.

[14] Parga-Landa B, Hernández-Olivares F (1995). An analytical model to predict impact behaviour of soft armours. *International Journal of Impact Engineering*.

[15] Park HC, Park J, Goo NS, Yoon KJ, Lee JH (2004). Analysis of low-velocity impact on composite sandwich panels using an assumed strain solid element. *Key Engineering Materials*.

[16] Herup EJ (1996). *Low-velocity impact on composite sandwich plates [Dissertation]*.

[17] Davies GAO, Hitchings D, Zhang X Damage tolerance to low velocity impact of laminated composites.

[18] Johnson AF, Pickett AK Impact and crash modeling of composite structures: a challenge for damage mechanics.

[19] Zheng X, Goldberg RK, Binienda WK, Roberts GD LS-DYNA implementation of Polymer Matrix Composite Model under high strain rate impact.

[20] Shultz C, Peters J (1998). *Bird Strike Simulation Using LS-DYNA*.

[21] Hussainova I, Schade K-P, Tisler S (2006). Dynamic coefficients in impact mechanics. *Proceedings of the Estonian Academy of Sciences*.

[22] Johnson AF, Pentecôte N Modelling impact damage in double-walled composite structures.

[23] Reyes G (2008). Static and low velocity impact behavior of composite sandwich panels with an aluminum foam core. *Journal of Composite Materials*.

[24] Elder DJ, Thomson RS, Nguyen MQ, Scott ML (2004). Review of delamination predictive methods for low speed impact of composite laminates. *Composite Structures*.

[25] Kelkar AD, Tate JS, Bolick R Introduction to low cost manufacturing of composite laminates.

[26] Bolick R, Kelkar AD Innovative composite processing By using HVartm©method.

[27] Chamis CC Simplified composite micro-mechanics for predicting microstresses.

